# Prevalence of abnormal glucose metabolism in atrial fibrillation: A case control study in 75-year old subjects

**DOI:** 10.1186/1475-2840-7-28

**Published:** 2008-09-28

**Authors:** Odd Erik Johansen, Ellen Brustad, Steve Enger, Arnljot Tveit

**Affiliations:** 1 Medical department, Asker and Baerum Hospital, PO Box 83, 1309 Rud, Norway

## Abstract

**Background:**

The prevalence of atrial fibrillation (AF) is increasing world wide and amongst factors that aggravate the risk is diabetes mellitus (DM), also in epidemic development.

However, although DM is a potentially modifiable risk factor for AF, few, if any, studies have explored the prevalence of undiagnosed dysglycaemia among subjects with AF or if duration of AF are related to parameters of glycaemia or dysglycaemia prevalence.

**Methods:**

In this case control study, amongst 75-year old subjects with and without AF, the prevalence of dysglycaemia, i.e., impaired fasting glycaemia, impaired glucose tolerance or DM, according to World Health Organisation criteria was assessed by a 75-g oral glucose tolerance test (OGTT).

**Results:**

Prevalence of undiagnosed DM among the 108 subjects (male/female 73/35, BMI 25.4 ± 3.2) without and the 46 (male/female 34/12, BMI 25.3 ± 3.7) with AF (median AF duration five years) where 3.7% and 13.0%, respectively (p = 0.031, Odds ratio (OR) 3.86 (95% Confidence interval [CI]: 1.01, 16.25)) whereas the overall prevalence of dysglycaemia (prediabetes and DM) where similar (respectively 43.5% and 39.1%, p = 0.46, OR 0.83 [95% CI: 0.41, 1.69]). Patients with AF duration ≥ 5 years had however a higher dysglycaemia prevalence (61.1% [DM 22.2%, prediabetes 38.9%]) as compared to AF duration < 5 years (25% [DM 7.1%, prediabetes 17.9%], p = 0.0014, OR 4.7 [95% CI: 1.30, 16.90]) or no AF (p = 0.17, OR 2.04 [95% CI: 0.73, 5.66]). There was also a significant correlation between the duration of AF and HbA1c (r = 0.408, p = 0.005) and fasting glucose levels (r = 0.353, p = 0.016).

**Conclusion:**

AF is associated with chronic hyperglycaemia amongst 75-year old subjects. Prediabetes and DM should be pro-actively assessed if AF duration ≥ 5 years.

## Background

The prevalence of atrial fibrillation (AF) doubles with each decade of age in the elderly [1], which is attributed to accompanying cardiac abnormalities occurring [2], although also linked to other independent AF risk factors (some which also are increasing with age), e.g., prior AF, male sex, depressed ejection fraction, hypertension, atrial enlargement, valvular heart surgery, chronic renal insufficiency, rheumatic heart disease, obesity and inflammation [1,3,4]. Today, AF affects about 8% of subjects aged 65 or older [5], and due to the ageing population, a 2.5 fold increase in AF prevalence during the next 50 years has been suggested [6].

One concern added to this is that one potentially modifiable risk factor for AF, namely diabetes mellitus (DM), also is occurring with growing prevalence globally [7]. DM is a relative strong AF risk factor and renders patients approximately 1.4–2.1 times more prone to develop AF [8-10]. Hence, the growing prevalence of DM could further aggravate the projected increase in AF prevalence [6].

Although DM account for 10–25% of AF patients [8-10], and the fact that the increased incidence of AF is not only limited to those with established or new-onset DM [10], but even also affects patients, at least during cardiac surgery, with lesser degrees of hyperglycaemia [11], few studies have investigated whether interventions reducing the blood glucose levels could reduce the AF burden. However, some reports supports that this could be the case, e.g. a 1600 kcal/day diet and walking three times/day for more than 30 min have been reported to eliminate paroxysmal AF after six months in an older women [12] and lowering blood glucose with insulin during cardiac surgery have been associated with lower AF incidence [13], although debated [14]. Regression of paroxysmal AF has also been reported with rosiglitazone treatment (a peroxisome proliferator receptor activator indicated for the treatment of hyperglycaemia in type 2 DM) in two patients [15].

Hence the detection of glucose disturbances in AF for these reasons could be of importance. Perhaps more importantly though, is that hyperglycaemia among subjects with AF contributes to an added risk for stroke [16] and convincing data now support aggressive glucose control to prevent stroke in patients with DM [17].

However, few, if any, studies have investigated the prevalence of undiagnosed abnormal glucose metabolism in patients with AF, in contrast to investigations among patients with cardiovascular (CV) disorders [18,19] where this consistently have been reported higher than 50%, twice that of age matched controls [18].

Given the association between hyperglycaemia as a risk factor for AF and hyperglycaemia being modifiable, we assessed the glycaemic status (as defined according to an oral glucose tolerance test [OGTT]) among older patients with AF and age-matched controls without, all being previously undiagnosed with abnormal glucose metabolism. Furthermore, we investigated whether the duration of AF could be related to parameters of glycaemia or dysglycaemia prevalence.

## Methods

This study was conducted at the Asker and Baerum Hospital in Eastern Norway as a sub-study of the Asker and Baerum Atrial Fibrillation study [20], an epidemiological study of AF in all 75-year old men and women (born in the year 1930) who were permanent residents of the coverage area of hospital (n = 1117), where 82% (n = 916) attended. In the present case-control study we assessed the prevalence of previously undiagnosed abnormal glucose metabolism among subjects with and without AF through an OGTT. In addition, other glucometabolic parameters were measured in plasma, as well as measurement of anthropometric parameters and blood pressure. Collection of medical history and current use of medication were recorded from questionnaires and interview.

The participants for this study were consecutively recruited when identified with AF on a first-come first-serve basis, and for each subject with AF that agreed to participate, the next two subjects in sinus rhythm of the same gender were recruited as control subjects. Exclusion criteria were neurological deficits or other medical conditions resulting in incapability of performing an OGTT, a previous diagnosis of prediabetes, e.g., impaired fasting glycaemia (IFG) or impaired glucose tolerance (IGT), DM or a history of gestational DM, an unstable medical condition or an acute or chronic illness known to influence OGTT (e.g. infection).

The protocol was approved by the Regional Committee for Medical Research Ethics, Norway and by the Norwegian Data Inspectorate. Each subject gave informed written consent before participating in the study. No compensation was given for the participation in the study.

### Glucometabolic parameters and definition of the metabolic syndrome

An OGTT with ingestion of 75 g. glucose dissolved in 300 ml water flavoured with citric acid was performed according to WHO standards, but with the supplement of a 1 hour (h) – glucose measurement. Blood glucose during the OGTT was analyzed immediately in capillary whole blood (photometer; HemoCue, Ängelholm, Sweden).

HbA1c was analysed by colorimetric and immunoturbidimetrical methods (COBAS INTEGRA system, Roche Diagnostics, Mannheim, Germany) in whole blood with an upper normal limit reference value of 6.2%.

Area under the curve (AUC) for the OGTT was calculated as a measure of glycaemic burden, and was assessed by calculating the incremental area from start to 1 h and from 1 h to 2 h [21].

Insulin was analyzed by radioimmunoassay using an antibody with no cross-reaction against proinsulin (Diagnostic System Laboratories, Webster, TX). The homeostasis model assessment for insulin resistance (HOMA-IR) was calculated in fasting conditions as plasma insulin (pmol/l) × blood glucose (mmol/l) × 1.13 (correction for plasma glucose)/135 [22]. The median (interquartile range) for HOMA-IR in the reference group (the 75-year old subjects without AF) were 3.56 (2.76–4.96).

In this study, subjects without normal glucose tolerance (NGT) are referred to as being dysglycaemic, or having an abnormal glucose metabolism, i.e. prediabetes (IFG and/or IGT) or DM according to World Health Organisation criteria [23]. Metabolic syndrome was defined according to the National Cholesterol Education Program's Adult Treatment Panel III classification [24].

### Lipid parameters

Serum levels of total cholesterol, high-density lipoprotein (HDL)-cholesterol and triglycerides (TG) were measured enzymatically on a Roche/Hitachi 917 analyzer (Roche Diagnostics). Low-density lipoprotein (LDL)-cholesterol was calculated using the Friedewald formula.

### ECG and classification of hypertension

AF was assessed by twelve lead ECG that were recorded in the supine position after 5 min rest as described previously [19]. In patients currently in sinus rhythm, but with a history of AF, supplementary information was retrieved from hospital and general practitioners' records. Duration of AF was calculated based on year since first physician's diagnosis of AF.

Blood pressure was measured in the supine position after 10 min rest. If the initial blood pressure was greater than 160/95 mm Hg, a repeat measurement was done, and the lowest measurement was registered. Hypertension was defined as systolic/diastolic blood pressure > 140/90 mmHg or current use of any antihypertensive medication.

### Blood sampling protocol

Peripheral venous blood was drawn between 8 and 10 AM after an overnight fast and serum and plasma were collected as previously described [18]. The samples were analysed within 24 hours (routine chemistry, lipid and glucose parameters) or stored at -70°C until analysis (insulin).

### Statistics

Sample sizes where calculated based on an expected prevalence of undiagnosed dysglycaemia in the AF group of 50% [18,19] and in the controls of 25% [15]. Our null hypothesis was that there should be no difference in this prevalence between subjects with AF and controls. As we wanted a statistical power of 80% and an alpha error level of 5%, we then needed at least 45 individuals in each group. Data analysis was performed using SPSS statistical software version 14.0.1 for Windows (SPSS Inc.). Results for continuous variables are presented as mean and standard deviation unless otherwise stated. Between-group statistical analysis of continuous parameters utilized student's t-test or one-way ANOVA. Correlation coefficients are univariate derived using Spearman's correlation. Categorical variables are presented as counts or proportions (%) and statistical comparisons of these parameters were carried out by chi-squared test or the Fischer exact test. Odds ratio (OR) with 95% confidence interval (CI) was estimated for dysglycaemia and impact of congestive heart failure and valve surgery on dysglycaemia prevalence and atrial fibriallation duration was investigated in a logistic regression analysis. P-values were two-sided and considered significant when < 0.05.

## Results

We recruited 61 subjects with AF and 126 without of whom 13 had a previous diagnosis of DM (seven with AF and six without [p = 0.091]) and were excluded from the analysis. Hence, in total 54 subjects with AF and 120 subjects without AF were eligible. Among these, 154 agreed to perform the OGTT of whom 46 (30.3%) had AF (paroxysmal in 15 [32.6%], persistent or chronic in 26 [56.5%] and newly diagnosed in five [10.9%]). The median AF duration was five years (min 0, max 28).

All study participants were living in their own home, i.e. none were institutionalised, and further background characteristic and use of medications are given in table 1. As outlined, 3/4 of the subjects with AF received warfarin, while only four subjects without AF received this. This latter was due to previous heart valve surgery (n = 1), deep vein thrombosis (n = 1) and myocardial infarction (n = 2).

**Table 1:** Background data for the 75-year old study participants (n = 154).

	Atrial fibrillation	No atrial fibriallation	Chi-square/df/p or t/d.f/p
Gender (male/female)	34 (31.8%)/12 (25.5%)	73 (68.2%)/35 (74.5%)	0.608/1/0.44
BMI (kg/m^2^)	25.3 ± 3.7	25.4 ± 3.2	-0.176/145/0.86
Waist (cm)	93.5 ± 11.7	91.6 ± 10.9	0.936/151/0.35
SBP (mmHg)	143 ± 21	147 ± 16	-1.169/152/0.24
DBP (mmHg)	82 ± 10	81 ± 9	1.069/152/0.29
*Concomitant disorders, n (%)*			
Hypertension	38 (82.6%)	83 (76.9%)	0.635/1/0.43
MI	6 (13.0%)	11 (10.2%)	0.268/1/0.60
HF	7 (15.2)*	0 (0.0%)	< 0.001 (Fisher)
Valve surgery	3 (6.5%)*	1 (0.9%)	0.046 (Fisher)
CABG/PCI	6 (13.0%)	8 (7.4%)	1.240/1/0.27
Cerebrovascular disease	5 (10.9%)*	2 (1.9%)	0.025 (Fisher)
*Medication, n (%)*			
Warfarin	33 (76.1%)*	4 (3.7%)	< 0.001 (Fisher)
Acetylic acid	3 (6.5%)	33 (30.6%)*	0.001 (Fisher)
Digitoxin	8 (17.4)*	0 (0%)	< 0.001 (Fisher)
Verapamil	10 (21.7)*	2 (1.9)	< 0.001 (Fisher)
Other Ca2^+ ^blockers	4 (8.7)	11 (8.7)	1.000 (Fisher)
Beta blockers	14 (30.4%)*	16 (14.8%)	5.018/1/0.03*
ACE inhibitor	12 (26.1%)*	13 (12.0%)	4.683/1/0.03*
AT-II antagonist	13 (28.3%)*	13 (12.0%)	6.051/1/0.01*
Diuretics	12 (26.1%)*	5 (4.6%)	15.125/1/<0.001*
Statins	18 (39.1)*	19 (17.6)	8.198/1/0.01*
*Laboratory parameters*			
Total leukocyte count (10^9^)	6.0 ± 1.3	6.2 ± 1.6	-0.52/140/0.61
Creatinine (μmol/l)	84 ± 21	80 ± 23	0.93/137/0.35
Total cholesterol (mmol/l)	5.2 ± 1.0*	5.6 ± 1.1	-2.326/140/0.02*
Triglycerides (mmol/l)	1.1 ± 0.8	1.1 ± 0.6	0.162/140/0.87
HDL-cholesterol (mmol/l)	1.7 ± 0.6	1.7 ± 0.5	0.443/140/0.66
LDL-cholesterol (mmol/l)	2.9 ± 0.9*	3.4 ± 1.0	-2.713/139/0.01

*: p ≤ 0.05. Abbreviations: BMI – body mass index, SBP – systolic blood pressure, DBP – diastolic blood pressure, MI – myocardial infarction, HF – heart failure, CABG – coronary artery by-pass graft, PCI – percutaneous coronary intervention, Ca^2+^-calcium, ACE – angiotensin converting enzyme, AT-II – angiotensin II, HDL – high density lipoprotein, LDL – low density lipoprotein.

In total six previously undiagnosed cases of DM (13.0%) were detected among subjects with AF as compared to four (3.7%) among those without AF (p = 0.031, OR 3.86 [95% CI: 1.01, 16.25], table 2), whereas no differences in the prevalence of prediabetes (IFG/IGT) were found. Hence, the total prevalence of dysglycaemia among subjects with AF *overall*, where similar as among those without AF (respectively 39.1% and 43.5%, p = 0.46, OR 0.83 [95% CI: 0.41, 1.69]). This was also the case for the metabolic syndrome (41.3% among those with and 31.5% among those without AF, p = 0.24).

**Table 2:** Glucometabolic parameters, OGTT results and prevalence of the metabolic syndrome in subjects with and without atrial fibrillation.

	Atrial fibrillation	No atrial fibriallation	Chi-square/df/p or t/d.f/p
N	46	108	
HbA1c (%)	5.9 ± 0.3*	5.7 ± 0.4	1.953/139/0.05
Insulin (pmol/l)	98 ± 67	90 ± 39	0.887/120/0.38
HOMA-IR	4.35 ± 3.4	4.0 ± 1.9	0.730/120/0.47
Fasting glucose (mmol/l)	5.3 ± 0.6	5.3 ± 0.6	0.156/152/0.88
Delta 1-h glucose (mmol)l)	4.3 ± 2.2	3.9 ± 1.9	-1.299/152/0.20
Delta 2-h glucose (mmol/l)	2.0 ± 2.2	1.4 ± 1.6	-0.167/152/0.10
AUC Baseline-60 minutes	129.7 ± 65.6	116.1 ± 57.2	-1,299/152/0.20
AUC 60–120 minutes	193.2 ± 102.0*	161.1 ± 85.5	-2,010/152/0.05
OGTT results and the metabolic syndrome, n (%)			
NGT	28 (60.9%)	61 (56.5%)	6.143/2/0.46
Prediabetes	12 (26.1%)	43 (39.8%)	
DM	6 (13.0%)	4 (3.7%)	
Metabolic syndrome	19 (41.3%)	34 (31.5%)	1.379/1/0.240

*: p ≤ 0.05 Abbreviations: HbA1c – Haemoglobin A1c, HOMA-IR – homeostasis model assessment for insulin resistance, h – hour, AUC – area under the curve, OGTT – oral glucose tolerance test, NGT – normal glucose tolerance, DM – diabetes mellitus

**Figure 1:**
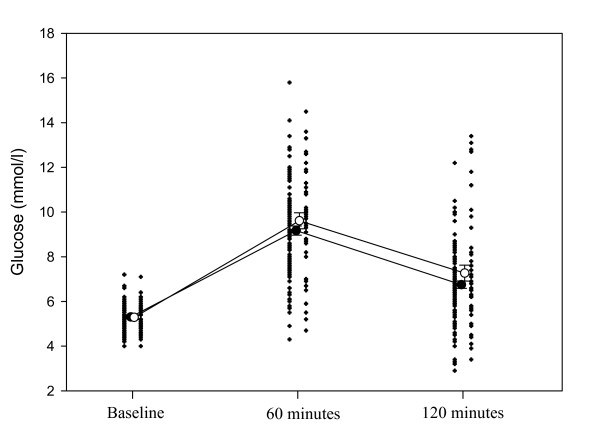
**Glucose values of the OGTT before and at 1 and 2 hours**. The figure indicates mean ± standard error glucose values among subjects with AF (white circles) and without AF (black circles) as well as individual results. The dots to the right of the circles are the results in the AF group whereas dots to the left indicate results for the non-AF group. Abbreviation: SE: standard error, OGTT: oral glucose tolerance test.

HbA1c were however higher among those with AF (5.9% vs 5.7%, p = 0.05) and there was a significant higher incremental AUC between the 1^st ^and 2^nd ^h of the OGTT (193.2 vs 161.1, p = 0.05), as well as a trend towards higher glucose levels at 1 h and 2 h during the OGTT (Figure 1).

**Figure 2:**
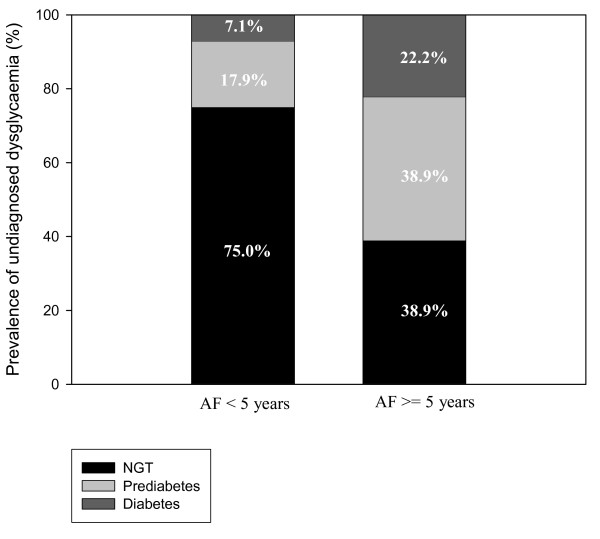
**Undiagnosed dysglycaemia according to duration of AF**. Prevalence of undiagnosed dysglycaemia in subjects with AF < 5 years and in subjects with AF ≥ 5 years.

In the AF group (Figure 2), subjects with AF duration ≥ 5 years (median value) had a statistical significant higher prevalence of abnormal glucose metabolism (61.1% [DM 22.2%, prediabetes 38.9%], OR 4.7 [95% CI: 1.3, 16.9]) as compared to those with AF duration < 5 years (25% [DM 7.1%, prediabetes 17.9%]) (p = 0.0014). This prevalence tended even to be higher than among the 75-year old subjects without AF (p = 0.17, OR 2.04 [95% CI: 0.73, 5.66]). This association was further supported by a significant positive correlation between the duration of AF and HbA1c (r = 0.408, p = 0.005) as well as fasting glucose levels (r = 0.353, p = 0.016), although no significant relation was seen for 1 h glucose, 2 h glucose, fasting insulin levels or HOMA-IR.

Interestingly, this increased prevalence among subjects with AF ≥ 5 years was not accompanied by a significant increased prevalence of the metabolic syndrome (without AF 31.5%, AF < 5 years 35.7%, AF ≥ 5 years 50.0%, p = 0.306), HOMA-IR (3.99, 4.01 and 5.16), use of CV medications or concomitant CV complications, although the prevalence of heart failure was higher in the above median group (0.0%, 3.6%, 33.3%, p < 0.001). In a logistic regression analysis however, neither heart failure nor valve surgery had an impact on the dysglycaemia prevalence according to median AF duration.

In the AF group, among those classified as having NGT, prediabetes or DM, mean AF duration (min, max) also differed significantly (p = 0.013), were this respectively were 4 ± 4 (0, 14), 9 ± 8 (0, 28) and 9 ± 6 (3, 20) years (p = 0.016). In a post-hoc (Bonferroni) analysis the differences reached statistical significances in the prediabetes vs the NGT group (p = 0.046) while a borderline significance was found between NGT vs DM (p = 0.096).

## Discussion

In this study we found an increased glycaemic burden among 75-year old subjects with AF as compared to matched subjects without AF and that the prevalence of previously undiagnosed prediabetes or DM among subjects in whom AF duration ≥ 5 years were high (61.1%), in fact at comparable rate as in patients with ischemic CV complications [18,19]. Hence, our findings suggest that undiagnosed abnormal glucose metabolism should pro-actively be investigated in older subjects with AF duration ≥ 5 years.

Chronic hyperglycaemia may contribute to the AF burden [8-11] in several ways, and one as recently described, could be through the activation of the AGE (advanced glycation endproduct) – RAGE (receptors for AGE) system and the up regulation of circulating tissue growth factors (CTGF) that may promote atrial structural remodelling [25]. A detection of dysglycaemia could therefore be of importance if a reduction in glycaemic burden could modulate the AF burden or transistion rate. Although this remains to be further proven, some, yet limited, literature in fact yields support [12,13,15].

Equally, or more, important though is that further glycaemic deterioration could be prevented by pharmacological or non-pharmacological means [26]. The glycaemic deterioration leads to an aggravation of risk for CV complications, which among subjects with AF, stroke is of particular concern [16,17], underscored by recommendation of AF to strongly be considered in patients with DM who present with stroke and no definite cause [9]. With the increasing prevalence of both DM and AF worldwide [5-7], especially among the elderly, an increase in the number of strokes and hospital admissions in this population is likely, thereby providing a large burden on the health care.

One potential reason for that long standing AF increases prevalence of an abnormal glucose metabolism could be related to an activation of inflammation, an early manifestation of AF as well as DM, hence potentially also being a common soil for this association. This was first underscored in a study relating AF and inflammation in a burden dependent manner, i.e., patients with persistent AF had higher levels of CRP than subjects with paroxsystic or no AF [3]. Interestingly, this also seems to be the case for dysglycaemia [18]. Given that chronic inflammation mediate its effect over time, it is also plausible that inflammation associated with AF over time could have an adverse impact on glucose metabolism.

Another possibility for that AF increase the glycaemic burden is that AF may render subjects with poorer exercise capacity [27] hence restricting them in reaching advised levels of leisure time physical activity. Reduced physical activity is a well established risk factor for both diabetes and prediabetes [26], but moreover, could also contribute to an accelerated conversion rate from prediabetic states to DM [26].

### Limitations

This study has some limitations a part from the relatively small sample size and the exclusive inclusion of 75-year old subjects, although the latter also could be considered strength of the study. Differences in vascular co-morbidity found between the groups make it harder to interpret the relative contribution of AF respective CV complications on dysglycaemia. The different rates of beta-blocking agent use and diuretics could also influence the OGTT-results adversely. However, the analyses of subjects with AF according to median AF duration revealed no differences in the use of CV medications or concomitant CV complications (only heart failure), yet a significant difference in the prevalence of previously undiagnosed dysglycaemia was noted.

In this study a higher use of statins and compounds blocking the renin-angiotensin system were found in the AF group. Such use have been associated with lower prevalence of DM [28], hence this could reduce the impact of AF on chronic hyperglycaemia. However, the glucose lowering abilities of these medications are probably small, although the AF group had decreased levels of LDL-cholesterol as well as a tendency to lower blood pressure.

## Conclusion

In conclusion, this study suggests that undiagnosed DM and prediabetes among older subjects with AF should pro-actively be assessed if duration of AF ≥ 5 years. Further studies are needed to clarify how long standing AF may in some way induce chronic hyperglycaemia, and if glucose lowering strategies reduces the AF burden.

## Competing interests

The authors declare that they have no competing interests.

## Authors' contributions

OEJ and AT conceived the study and OEJ drafted the manuscript, EB and SE were involved in the coordination and data acquisition, OEJ and AT interpreted the results and OEJ performed the statistical analysis of the data presented, OEJ, EB, SE and AT critically reviewed the study for important intellectual content. All authors approved the final version of the manuscript.
